# Face Recognition, Musical Appraisal, and Emotional Crossmodal Bias

**DOI:** 10.3389/fnbeh.2017.00144

**Published:** 2017-08-02

**Authors:** Sara Invitto, Antonio Calcagnì, Arianna Mignozzi, Rosanna Scardino, Giulia Piraino, Daniele Turchi, Irio De Feudis, Antonio Brunetti, Vitoantonio Bevilacqua, Marina de Tommaso

**Affiliations:** ^1^Human Anatomy and Neuroscience Lab, Department of Environmental Science and Technology, University of Salento Lecce, Italy; ^2^Department of Psychology and Cognitive Sciences, University of Trento Trento, Italy; ^3^Santa Chiara Institute Lecce, Italy; ^4^Department of Electrical and Information Engineering, Polytechnic University of Bari Bari, Italy; ^5^Department of Medical Science, Neuroscience, and Sense Organs, University Aldo Moro Bari, Italy

**Keywords:** music cognition, face recognition, N170 ERP, emotional salience, crossmodal integration, emotional biases, musical appraisal

## Abstract

Recent research on the crossmodal integration of visual and auditory perception suggests that evaluations of emotional information in one sensory modality may tend toward the emotional value generated in another sensory modality. This implies that the emotions elicited by musical stimuli can influence the perception of emotional stimuli presented in other sensory modalities, through a top-down process. The aim of this work was to investigate how crossmodal perceptual processing influences emotional face recognition and how potential modulation of this processing induced by music could be influenced by the subject's musical competence. We investigated how emotional face recognition processing could be modulated by listening to music and how this modulation varies according to the subjective emotional salience of the music and the listener's musical competence. The sample consisted of 24 participants: 12 professional musicians and 12 university students (non-musicians). Participants performed an emotional go/no-go task whilst listening to music by Albeniz, Chopin, or Mozart. The target stimuli were emotionally neutral facial expressions. We examined the N170 Event-Related Potential (ERP) and behavioral responses (i.e., motor reaction time to target recognition and musical emotional judgment). A linear mixed-effects model and a decision-tree learning technique were applied to N170 amplitudes and latencies. The main findings of the study were that musicians' behavioral responses and N170 is more affected by the emotional value of music administered in the emotional go/no-go task and this bias is also apparent in responses to the non-target emotional face. This suggests that emotional information, coming from multiple sensory channels, activates a crossmodal integration process that depends upon the stimuli emotional salience and the listener's appraisal.

## Introduction

The wide discussion of recent research on the interaction between music and emotion addresses various issues, mainly those relating to comparisons between emotional processing and sensory experience, and the definition of music a process of “sense making” that involves and influences aspects of perception and cognition, as posited in a joint model of embodied mind (Reybrouck, 2005; Reybrouck and Brattico, 2015; Schiavio et al., 2016). Pioneering research on the crossmodal integration of visual and auditory perception suggests that evaluations of emotional information in one sensory modality may tend toward the emotional value generated in another (de Gelder and Vroomen, 2000; Logeswaran and Bhattacharya, 2009; Balconi and Carrera, 2011). Ad example, a realistic model able to explain emotional recognition process and crossmodal integration is the model of Balconi (Balconi and Carrera, 2011). This model is based on an experiment of a face recognition task interfaced, in crossmodal condition, with prosody, and analyzed through P2 ERP. The model highlights how an early ERP component (i.e., P2) can be considered a cognitive marker in multisensory processing. Thus, the emotion produced by musical stimulation, as could be prosody in the previous model, may influence the stimuli perception of stimuli, presented in other sensory modalities, through a top-down process (Sekuler et al., 1997; Jolij and Meurs, 2011; Wong and Gauthier, 2012). Different musical genres can also modulate arousal and others psychophysiological parameters eliciting different emotions (Schellenberg, 2005; Caldwell and Riby, 2007; Sammler et al., 2007; Fritz et al., 2009; Ladinig and Schellenberg, 2012; Schellenberg and Mankarious, 2012; Kawakami et al., 2014; Bhatti et al., 2016). For example, Baumgartner and colleagues investigated the psychophysiological effect of the interaction between emotional visual images, music, and a crossmodal presentation (music and images; Baumgartner et al., 2006). More intensely perceived emotions emerged in the crossmodal condition, and this was accompanied by predominant alpha band activity in EEG.

It has been proposed that music primes emotional responses to information in the visual domain (Logeswaran and Bhattacharya, 2009). Logeswaran and Bhattacharya demonstrated that musical priming (positive or negative) can modulate perceptions of emotional faces. In their study, participants were asked to rate the emotional salience of the faces, and the results demonstrated the existence of a crossmodal priming effect. Happy faces were rated as happier when they were presented after a happy piece of music and vice versa. The priming effect of music is evident with neutral targets. Analysis of Event-Related Potential (ERP) components showed that the N1 response to neutral faces increases when stimulus presentation is preceded by happy music than when it was preceded by sad music. Previous studies have observed an increased N1 component in the auditory cortex during simultaneous presentation of an emotionally congruent face (i.e., face–voice pairs; Pourtois et al., 2002). The N1 component was distributed over the frontal regions, suggesting the involvement of top–down psychophysiological mechanisms (Zanto et al., 2011; Gilbert and Li, 2013). Moreover, the perception of music is affected by the listener's emotional and cognitive state (Kawakami et al., 2013, 2014). Many studies have highlighted differences between the cognitive processing and cortical responses of musicians and non-musicians (Pantev et al., 1998; Brattico et al., 2010; Müller et al., 2010; Pallesen et al., 2010; Herholz and Zatorre, 2012; Proverbio et al., 2013).

Recent studies suggest that musical stimulation may interact with fatigue and motor activity, thereby affecting the motivation of individuals who are under intense physical stress (Bigliassi et al., 2016a,b). Music can modulate perception and cognition via a complex interaction between the perceptual and emotional characteristics of a musical stimulus and the physical (i.e., sex differences; Miles et al., 2016), psychophysiological, (Gosselin et al., 2007) and cognitive characteristics of the listener. Because of this interaction, the emotion invoked by music can result in biased responses (Chen et al., 2008). The aim of our study was to investigate how cross-modal perception—in this instance processing of emotional faces whilst performing a task that involves listening to music—varies with the subjective emotional salience of the music and with musical competence. This effect can be seen at cognitive and behavioral level, in decisions and appraisals, (Ellsworth and Scherer, 2003) and at motor level (in motor reaction time; Brattico et al., 2013). In fact, the motor and perceptual systems can be subject to early, top-down modulation induced by crossmodal stimulation, which can induce emotional bias, reflected at the behavioral level and in cortical responses (i.e., electrophysiological level). We also evaluated whether this bias could be modulated by the participant's appraisal of the musical stimulus (Brattico and Jacobsen, 2009) choosing an electrophysiological investigation of N170 ERP component. N170 ERP component is the most sensible ERP component able to be modulated in the Face Recognition Tasks (Eimer, 2000, 2011; Heisz et al., 2006; Kolassa et al., 2009; Ibanez et al., 2012; Leleu et al., 2015; Almeida et al., 2016). In particular, N170 is strictly linked to automatic processes (Heisz et al., 2006), instead of P2, that is a demonstrated cognitive marker in crossmodal cognition (Balconi and Carrera, 2011; Peretz, 2012). Still, in the condition in which music is perceived as a cognitive expertise, the emotional salience of the stimulus observed (i.e., face expression), may be affected by emotional bias, and this effect can be early observable through N170 modulations.

## Materials and methods

### Participants

Twenty-four participants were recruited in University and in Musical Conservatory, and were selected according to their musical skills. Twelve musicians, graduates in a Musical Conservatory, (5 men and 7 women; mean age = 29.8 years; *SD* ± 7.2) were compared to a group of 12 non-musicians, University students (graduated of the three-year degree and attending the specialist degree) without educational musical training (7 men and 5 women; mean age = 26.9 years; *SD* ± 4.5). The instruments played by the group of musicians included piano, guitar, trumpet, and trombone; one musician was a singer. All participants were right-handed, had normal hearing, and normal or corrected-to-normal vision. Participants provided written, informed consent to participation in accordance with the Helsinki Declaration. Participants did not receive any financial compensation. The local ethics committee (ASL Lecce, Apulia Region, Italy) approved the study.

### Materials

Participants performed an emotional go/no-go task (emo go/no-go), presented using E-Prime 2.0 (Richard and Charbonneau, 2009), during the EEG recordings.

The emotional go/no-go task (Schulz et al., 2007; Waters and Valvoi, 2009; Yerys et al., 2013) is a variant of the cognitive go/no-go task (Gomez et al., 2007) in which emotional information, measured through a decision-making process, is accompanied by a motor response. Generally, during an emo go/no-go task, the participant has to press the spacebar of a keyboard in response to an emotional face (neutral, angry, fearful, or happy). The choice of the face emotional expression depends on the task and on the process being investigated. The emo go/no-go task is a paradigm often used in ERP studies investigating a mismatch in response to stimulus salience (Jodo and Kayama, 1992; Smith et al., 2013; Moreno et al., 2014; Invitto et al., 2016). The N170 ERP component is the most sensitive in face recognition tasks (Eimer, 2000, 2011; Heisz et al., 2006; Blau et al., 2007). In this study, the computerized behavioral task required participants to press the spacebar when they identified a neutral face; EEG data were recorded whilst they were performing the task. Facial expressions were extracted from the NimStim Set of Facial Expressions (http://www.macbrain.org/resources.htm).

The NimStim Set is a collection of 672 images of the faces of 70 professional actors displaying various emotional expressions. The actors are of varying ethnicity and are represented in the same proportions by women and men. The collection consists of images of eight emotional facial expressions: fear, happiness, sadness, anger, surprise, disgust, neutral, and calm. In this experiment, we presented a sample of 64 images of fearful, happy and neutral faces, the expression categories were matched for sex and ethnicity.

In each condition the go-no-go task was accompanied by one of the following pieces from the classical piano repertoire:

- Chopin: Nocturne Op. 9 n. 1 and Nocturne Op. 9 n. 2.
- Mozart: Sonata in D major, K.V. 311.
- Albeniz: In Iberia, Rondeña.

Musical stimuli were delivered via two earphones, with a Windows 7 reproduction intensity of 60% (−6.4 dB), Conexant Smart Audio HD, Roland Sound Canvas, with a sampling rate of 48,000 Hz and 24-bit depth (system information: professional quality).

Each condition began with the listening of a musical piece, selected from the pieces above, whilst participants were performing the emo go/no-go task, looking at the emotional face displayed on the screen.

Participants rated the sadness and happiness each piece of music invoked using visual analog scales (VASs). The scales were administered immediately after each condition. The VASs consisted of a ten-centimeter line with the poles labeled 0 (absence of pleasure, sadness or happiness) and 10 (highest possible degree of pleasure, sadness, or happiness).

Each condition lasted approximately 500 s. Images of neutral (target), fearful and happy (non-target) faces were presented in pseudo-random order. Both target and non-target images were presented for 1,500 ms and the interstimulus interval was 1,500 ms.

Participants were instructed to sit so that there was a gap of about 75 cm between the front edge of the chair and the base of the computer screen. They had to listen to the pieces of classical music and respond to the presentation of neutral face on the screen by pressing the spacebar of the computer keyboard. At the end of each condition participants rated the emotions the accompanying music had elicited using the VASs described above.

### N170 ERP recording

EEGs were recorded from 64 active channels, mounted in an electrode cap according to the International 10–20-system. Signals were recorded through Brain Vision actiCHamp (Brain Products GmbH); the recording software was Brain Vision Recorder and the analysis software was Brain Vision Analyzer (Brain Products GmbH). Electrode impedance was kept below 15 kΩ. The EEG was amplified (band pass 0.1–40 Hz, 24 dB), with a sampling rate of 1000 Hz. Electrodes were referenced online to the FpZ. One electrode placed at the outer canthus of the right eye and used to monitor horizontal eye movements. Vertical eye movements and blinks were monitored by electrodes above and below the left eye. Trials contaminated by eye movements, amplifier conditioning, or other artifacts were rejected. The signal was filtered offline (0.01–50 Hz, 24 dB), and the threshold for artifact rejection was set at > |125|μV. The ocular rejection was performed through independent component analysis (ICA). The ERP epochs included a 100-ms pre-stimulus baseline period and a 500-ms post-stimulus segment. Separate averages were calculated for each facial expression (neutral, happy, and fearful) in each music condition (Albeniz, Mozart, and Chopin). The onset of ERP N170 peaks was estimated from grand average waveforms, according to the ERP latency definition (Heisz et al., 2006; De Vos et al., 2012; Smith et al., 2013). Peaks were automatically detected for all channels, using the global maxima in interval method (Giroldini et al., 2016).

## Data analysis and results

To investigate the role of the experimental manipulation on behavioral and psychophysiological data, we combined a linear mixed modeling with a decision-tree learning approach. Statistical analyses on linear mixed-models were performed with lme4, car, and lmertest packages supplied in the R environment whereas the decision-tree model was built by means of a tailor-made algorithm (Menolascina et al., 2007).

### Behavioral data

Independent-samples *t*-tests were used to analyze data from the three VASs for each condition (see Table 1).

**Table 1:** Independent-samples *t*-tests of VAS results.

**Musician**	**Emotion**	***t***	**df**	**Two-tailed significance**	**Mean VAS score**	
					**Musicians**	**Non-musicians**
Albeniz	Pleasant	2.138	22	0.044	9.09	7.23
Mozart	Happy	3.537	22	0.002	8.91	6.85
Chopin	Pleasant	2.690	22	0.013	9.27	7.46
Chopin	Sad	6.546	22	0.000	8.64	4.31

T-tests are conducted with respect to the Group variable (Non-Musicians vs. Musicians) over different emotional levels (Pleasant, Happy, Sad) and different Music (Albeniz, Chopin, Mozart). Note that T-value are computed with the t-statistics corrected for paired groups, df indicates the degree of freedom of the test, whereas p-values are computed considered alpha = 0.05

A repeated measures ANOVA was performed to analyze behavioral Reaction Time to neuter faces in the Emo Go/No-Go paradigm. The analysis considered Music (Albeniz, Chopin, Mozart) as within factor (3 Levels) and Group (2 Levels) as between factor. The model showed significant results in Group (*F* = 57.055, df = 1, *p* = 0.01), results just over the limits of statistical significance in Music condition (*F* = 2.947, df = 2, *p* = 0.053) and an interaction Music condition × Group (*F* = 3.012, df = 2, *p* = 0.049). The results showed a trend in higher response times in the musicians group, with a slower reaction time in Chopin session (Table 2).

**Table 2:** Mean of the behavioral reaction times (in millisecond) in response to neutral faces during the emo go/no-go task.

**Group**	**Reaction time**		
	**Albeniz**	**Chopin**	**Mozart**
Musicians	744.75	721.31	662.15
Non-Musicians	564.29	587.42	570.57

*Mean of reaction time in musicians is slower than in non-musicians group*.

### Psychophysiological data

The latency and amplitude of the N170 component were analyzed using separate linear mixed-effects models (LMMs) lme4 package (Bates et al., 2015) supplied as part of the R package (Bates et al., 2013, 2014). In both models, Group (musicians; non-musicians) and Music (Albeniz; Mozart; Chopin) were defined as fixed factors and participant and channel were coded as random effects. The interaction between Group and Music was also examined in the models. Sixty-one EEG electrodes were clustered into four main regions (ROIs): left anterior (L-Ant), right anterior (R-Ant), left posterior (L-Post) and right posterior (R-Post). Left and right were defined according to the standard international 10–20 system whereas anterior and posterior were defined according to the following rule: ANT (F, Fp, FC, FT, C, T, AF) and POST (TP, CP, P, PO, O; Frömer et al., 2012; Bornkessel-Schlesewsky and Schlesewsky, 2013) and as according the recent suggestions about the reduction of data dimensions (Luck and Gaspelin, 2017). To investigate potential regional differences, separate LMM analyses were run for reach ROI. In all these models, the Face variable was kept fixed at the Neutral emotional level (i.e., Target variable in the behavioral task, as described in the Materials section). To identify graphically the Regions of interest (ROIs), were processes through Analyzer a Pooling Elaboration with the creation of 4 New areas: Right Anterior (R-Ant), Right Posterior (R-Post), Left Anterior (L-Ant) and Left Posterior (L-Post).

#### N170 amplitude

**Figure 1:**
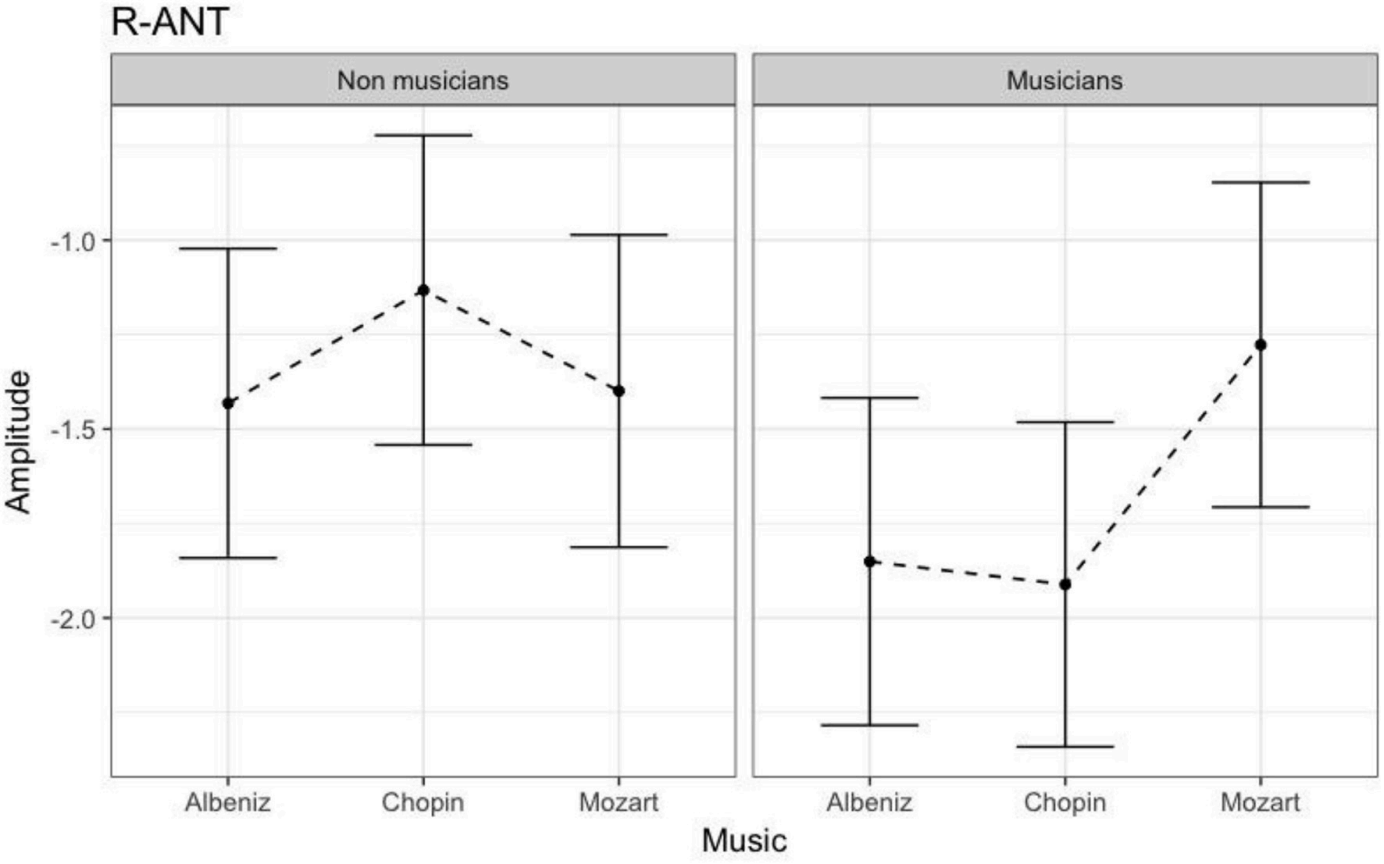
N170 amplitudes in non-musicians and musicians in R-ANT ROI (Right Anterior Region of Interest).

**Figure 2:**
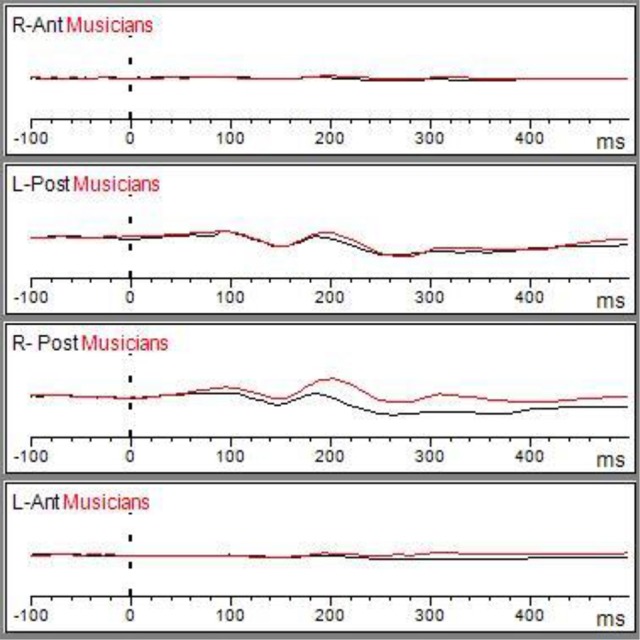
Matching ERP of Grand average elicited by the emo go/no-go face recognition task in non-musicians in (black line) and musician (red line) in right anterior, right posterior, left anterior, and left posterior regions. The Graphic of ROIs Regions has been performed through the channels pooling processing.

**Figure 3:**
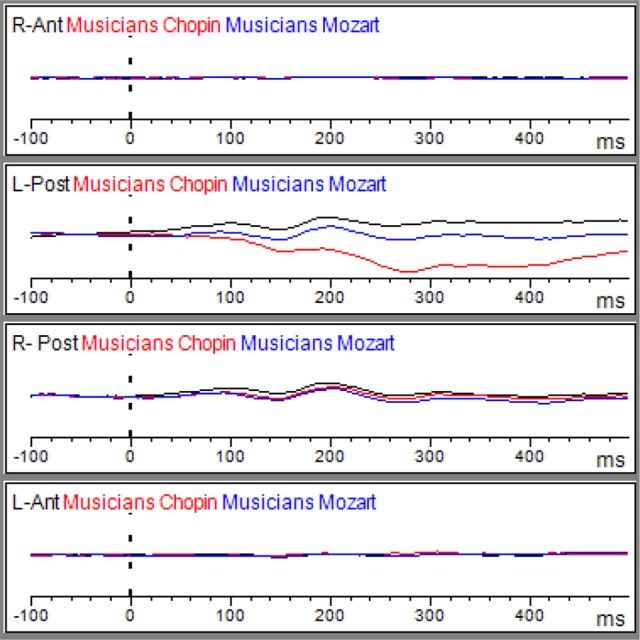
Grand average of the ERP components elicited by the emo go/no-go face recognition task in non-musicians in the Albeniz (black line), Chopin (red line), and Mozart condition (blue line) in right anterior, right posterior, left anterior, and left posterior regions.

**Figure 4:**
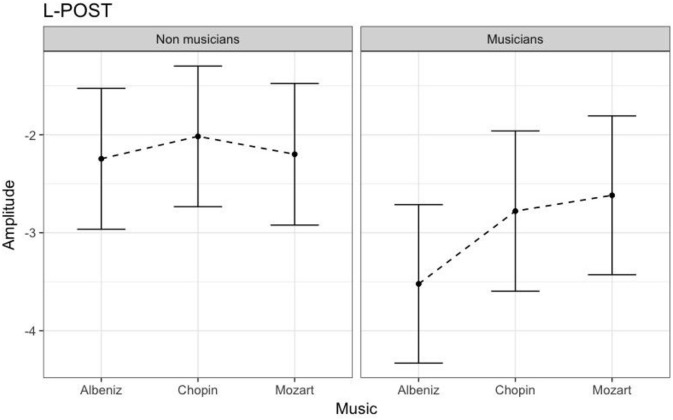
N170 amplitudes in non-musicians and musicians in L-POST ROI (Left Posterior Region of Interest).

**Figure 5:**
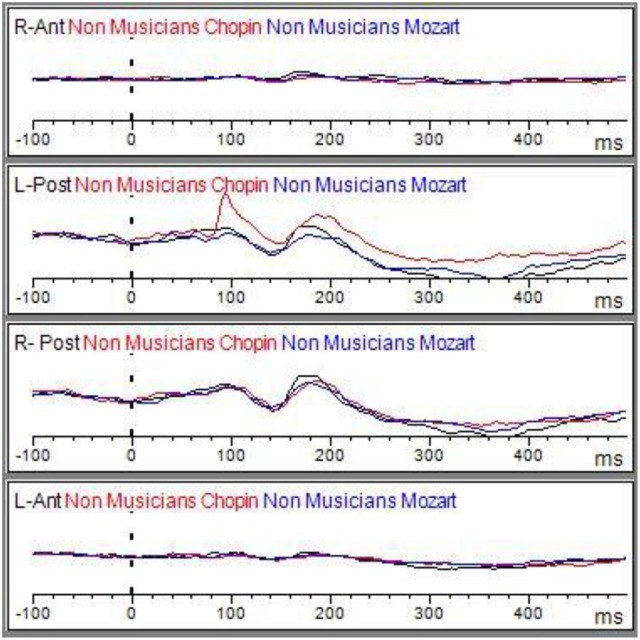
Grand average of the ERP components elicited by the emo go/no-go face recognition task in non-musicians in the Albeniz (black line), Chopin (red line), and Mozart conditions (blue line) in right anterior, right posterior, left anterior, and left posterior regions.

**Figure 6:**
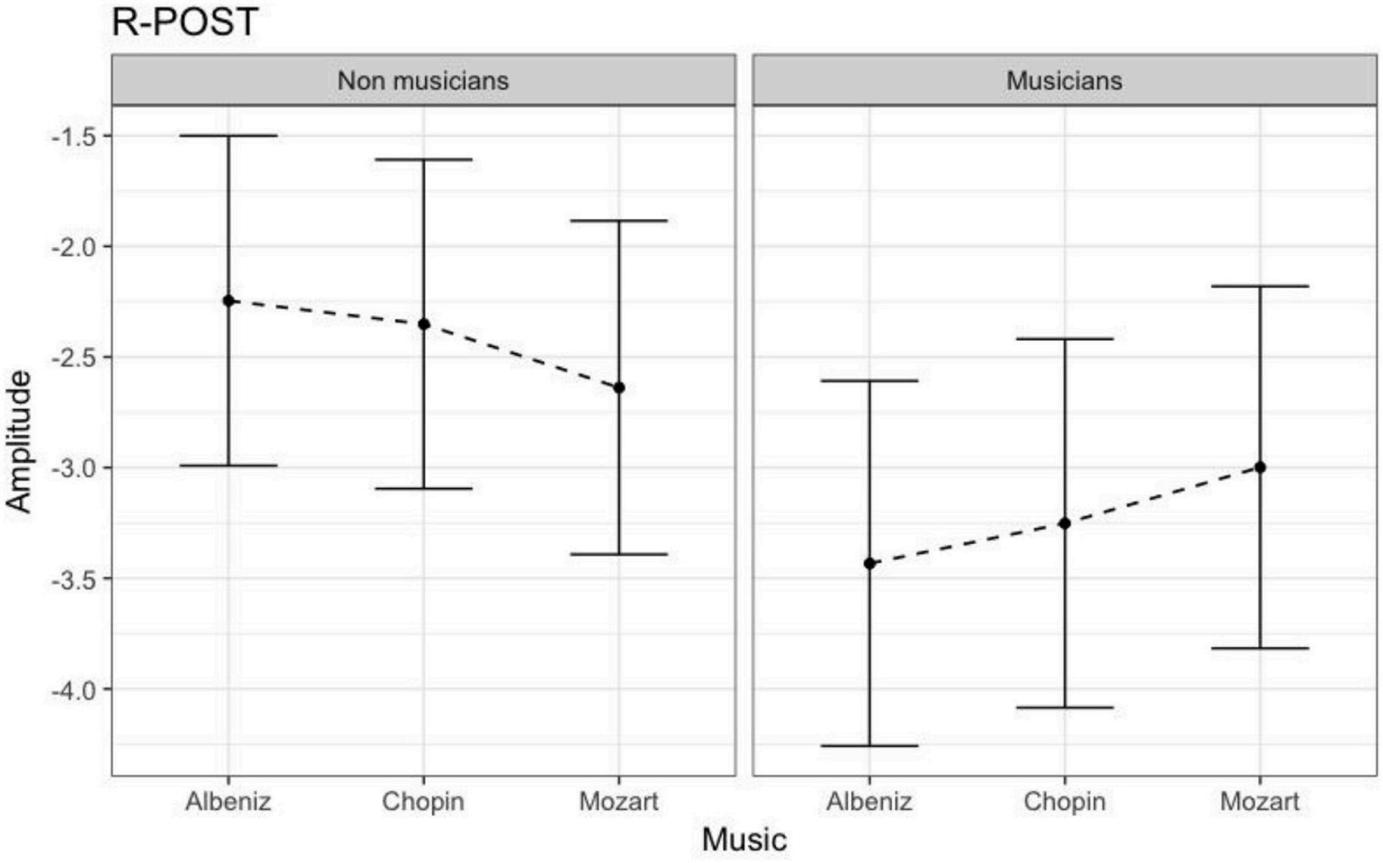
N170 amplitudes in non-musicians and musicians in R-POST ROI (Right Posterior Region of Interest). N170 amplitudes in non-musicians and musicians in R-POST ROI (Right Posterior).

**Figure 7:**
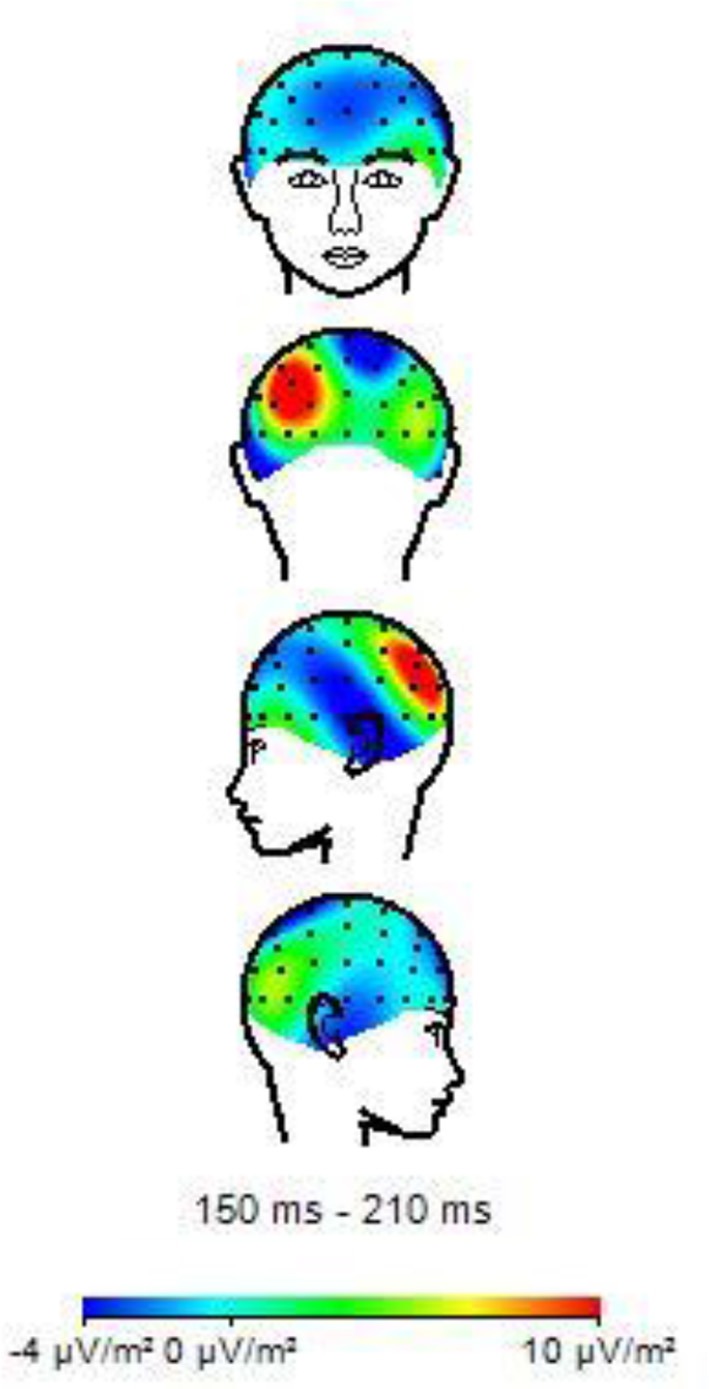
Topographies of N170 amplitude elicited by neutral facial expressions in non-musicians.

**Figure 8:**
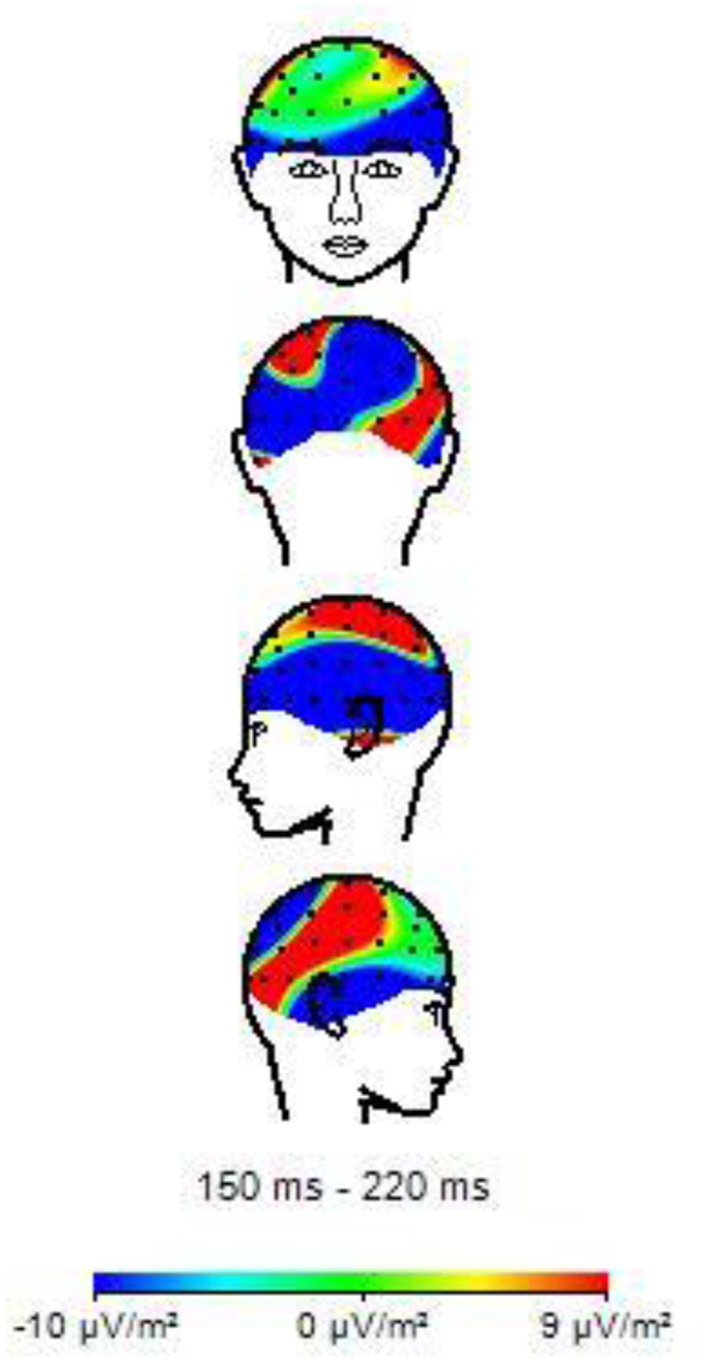
Topographies of N170 amplitude elicited by neutral facial expressions in musicians.

Table 3 shows the results of LMMs for N170 amplitude. In the L-Ant region there was no effect of Group or Music, although there was an interaction (*B* = −2.152, *t*_896_ = −2.152, *p* = 0.03). In the R-Ant region there were main effects of Group (*B* = −0.419, *t*_32_ = −2.11, *p* = 0.04; Figure 1) and Music (*B* = 0.299, *t*_906_ = 2.69, *p* = 0.007), reflecting ampler N170 in the musicians group and in the Chopin condition (Figure 2). There was also an interaction between Group and Music: musicians, in Chopin condition, revealed an increased amplitude (*B* = −0.360, *t*_910_ = −2.13, *p* = 0.03) and a decreased amplitude elicited in Mozart condition (*B* = 0.541, *t*_913_ = 3.16, *p* = 0.001; Figure 3). In the L-Post region there was an effect of Group (Figures 2, 4), reflecting increased N170 amplitude in musicians (*B* = −1.276, *t*_26_ = −6.13, *p* = 0.002). There was also a Group × Music interaction reflecting an increase in N170 amplitude in non-musicians during the Mozart condition (*B* = 0.857, *t*_537_ = 2.59, *p* = 0.009; Figure 5). In the R-Post region there was an effect of Group (Figure 6): N170 amplitude was greater in the musicians (*B* = −1.187, *t*_25_ = −2.16, *p* = 0.04; Figure 5), and Group × Music interaction: non-musicians showed an increase in N170 amplitude in the Mozart condition (*B* = 0.827, *t*_552_ = 2.42, *p* = 0.01). Respect these results, more negative components are visible through a Mapping imaging reconstruction in musicians vs. non-musicians (Figures 7, 8).

**Table 3:** Results of linear mixed-effects model: fixed effects for group and music on N170 amplitude.

**ROI**		**B (SE)**	***t***
L-Ant	Baseline	−1.423 (0.231)	−6.144
**Group**			
	Non-Musicians vs. Musicians	−0.175 (0.279)	−0.628
**Music**			
	Albeniz vs. Chopin	0.209 (0.137)	1.523
	Albeniz vs. Mozart	0.019 (0.141)	0.134
**Group** × **music**			
	Non-Musicians × Albeniz vs. Musicians × Chopin	−0.456 (0.212)	−2.152^*^
	Non-Musicians × Albeniz vs. Musicians × Mozart	0.070 (0.212)	0.331
R-Ant	Baseline	−1.431 (0.208)	−6.868
**Group**			
	Non-Musicians vs. Musicians	−0.419 (0.214)	−2.11^*^
**Music**			
	Albeniz vs. Chopin	0.299 (0.114)	2.701^**^
	Albeniz vs. Mozart	0.003 (0.110)	0.282
**Group** × **music**			
	Non-Musicians × Albeniz vs. Musicians × Chopin	−0.360 (0.168)	−2.133^*^
	Non-Musicians × Albeniz vs. Musicians × Mozart	0.541 (0.171)	3.161^**^
L-Post	Baseline	−2.245 (0.365)	−6.137
**Group**			
	Non-Musicians vs. Musicians	−0.687 (0.285)	−2.412^*^
**Music**			
	Albeniz vs. Chopin	0.123 (0.106)	1.155
	Albeniz vs. Mozart	0.024 (0.110)	0.225
**Group** × **music**			
	Non-Musicians × Albeniz vs. Musicians × Chopin	0.514 (0.328)	1.568
	Non-Musicians × Albeniz vs. Musicians × Mozart	0.857 (0.330)	2.596^**^
R-Post	Baseline	−2.245 (0.379)	−5.920
	**Group**		
	Non-Musicians vs. Musicians	−1.187 (0.550)	−2.157^*^
**Music**			
	Albeniz vs. Chopin	−0.105 (0.211)	0.617
	Albeniz vs. Mozart	−0.392 (0.225)	−1.741
**Group** × **music**			
	Non-Musicians × Albeniz vs. Musicians × Chopin	0.286 (0.339)	0.845
	Non-Musicians × Albeniz vs. Musicians × Mozart	0.827 (0.341)	2.422^*^

*Participants and EEG channels were treated as random effects; degrees of freedom of the model were calculated with the Satterthwaite approximation. L-Ant: left-anterior; R-Ant: right-anterior; L-Post: left-posterior; R-Post: right-posterior*.

* p < 0.05;

** *p < 0.01*.

#### N170 latency

**Figure 9:**
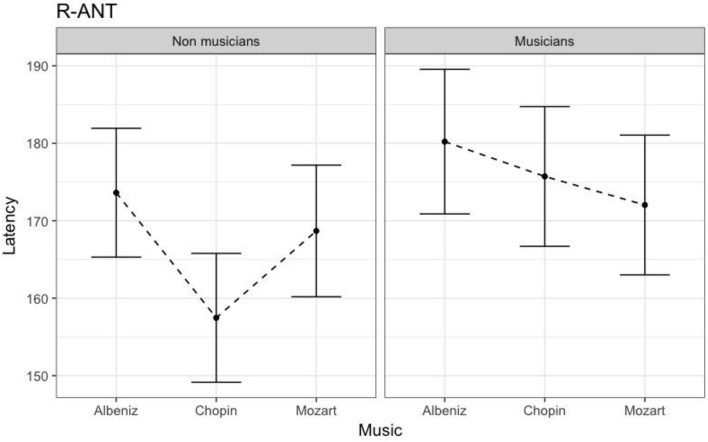
N170 Latency in non-musicians and musicians in R-ANT ROI (Right Anterior Region of Interest).

**Figure 10:**
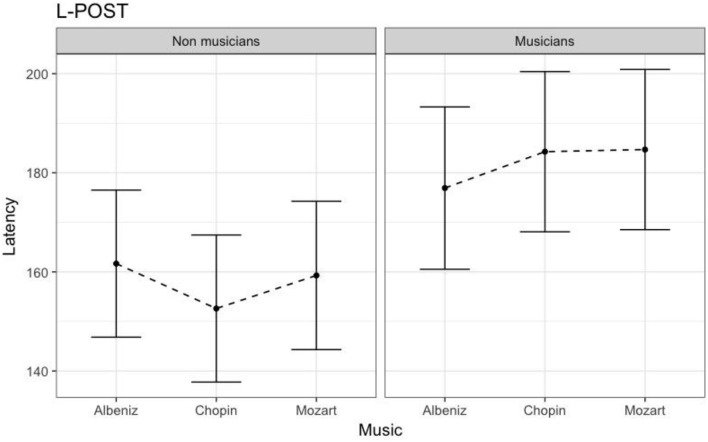
N170 Latency in non-musicians and musicians in L-POST ROI (Left Posterior Region of Interest).

**Figure 11:**
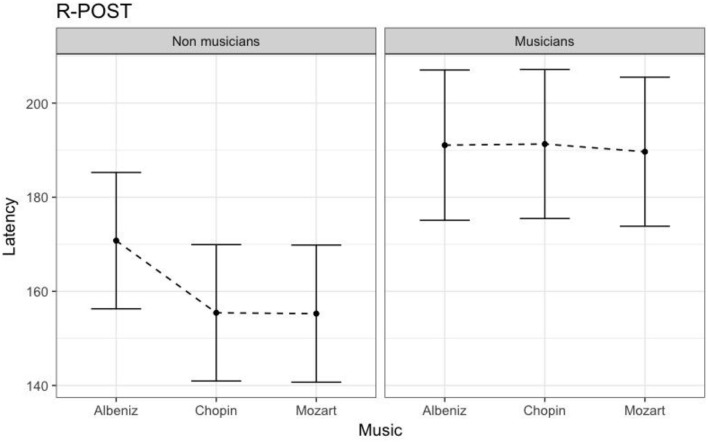
N170 Latency in non-musicians and musicians in R-POST ROI (Right Posterior Region of Interest).

Table 4 shows the results of LMMs for N170 latency. There were no effects of Group or Music in the L-Ant region. In R-Ant latencies (Figure 9) were shorter in the Chopin condition (*B* = −15.800, *t*_919_ = −4.25, *p* < 0.001), the opposite effect was found in L-Post (Figure 10), with slower latencies in the Chopin condition (*B* = −8.743, *t*_730_ = −2.16, *p* = 0.03). Finally, in the R-Post (Figure 11) region latencies were shorter in both the Chopin (*B* = −15.285, *t*_741_ = −4.88, *p* < 0.001) and Mozart (*B* = −15.543, *t*_743_ = −4.81, *p* < 0.001) conditions.

**Table 4:** Results of linear mixed-effects model: fixed effects for group and music on N170 latency.

**ROI**		**B (SE)**	***t***
L-Ant	Baseline	166.283 (34.783)	−0.609
**Group**			
	Non-Musicians vs. Musicians	9.178 (6.968)	1.317
**Music**			
	Albeniz vs. Chopin	−6.022 (3.935)	−1.531
	Albeniz vs. Mozart	1.214 (4.057)	0.299
**Group** × **music**			
	Non-Musicians × Albeniz vs. Musicians × Chopin	4.147 (5.958)	0.696
	Non-Musicians × Albeniz vs. Musicians × Mozart	4.111 (6.039)	0.681
R-Ant	Baseline	172.94 (4.38)	39.486
**Group**			
	Non-Musicians vs. Musicians	8.612 (6.320)	1.363
**Music**			
	Albeniz vs. Chopin	−15.80 (3.715)	−4.253^***^
	Albeniz vs. Mozart	−4.655 (3.829)	−1.216
**Group** × **music**			
	Non-Musicians × Albeniz vs. Musicians × Chopin	9.990 (5.624)	1.776
	Non-Musicians × Albeniz vs. Musicians × Mozart	−4.621 (5.701)	−0.811
L-Post	Baseline	101.215 (7.47)	21.569
**Group**			
	Non-Musicians vs. Musicians	16.991 (10.931)	1.554
**Music**			
	Albeniz vs. Chopin	−8.743 (4.041)	−2.163^*^
	Albeniz vs. Mozart	−2.552 (4.174)	−0.611
**Group** × **music**			
	Non-Musicians × Albeniz vs. Musicians × Chopin	14.881 (6.126)	2.430^*^
	Non-Musicians × Albeniz vs. Musicians × Mozart	9.282 (6.214)	1.493
R-Post	Baseline	169.701 (7.599)	22.332
**Group**			
	Non-Musicians vs. Musicians	21.699 (11.314)	1.918
**Music**			
	Albeniz vs. Chopin	−15.285 (3.216)	−4.889^***^
	Albeniz vs. Mozart	−15.543 (3.229)	−4.813^***^
**Group** × **music**			
	Non-Musicians × Albeniz vs. Musicians × Chopin	15.226 (4.739)	3.213^**^
	Non-Musicians × Albeniz vs. Musicians × Mozart	14.184 (4.808)	2.950^**^

*Participants and EEG channels were treated as random effects; degrees of freedom of the model were calculated with the Satterthwaite approximation. L-Ant: left-anterior; R-Ant: right-anterior; L-Post: left-posterior; R-Post: right-posterior*.

* p < 0.05;

** p < 0.01;

*** *p < 0.001*.

#### Assessing the gender effect

In order to evaluate whether the bias could be related to a gender effect, we proceed by comparing the fixed-effects structure of the previous linear-mixed models by adding and excluding the factor *gender* from the models. The results were evaluated in terms of model fit by using an information theory based approach (McElreath, 2016). To do so, for each ROI we considered two models: M0 (Simple model: excluding Gender variable) and M1 (Complex Model: including the Gender variable) and we fit the model via maximum likelihood. The BIC information criterion was then computed on the log-likelihood of the models along with the Vuong's statistic (Vuong, 1989; Merkle et al., 2016). Finally, asymptotic confidence intervals (CIs) on the BIC differences of the models (ΔBIC) were also computed. All the computations involved were performed by means of the nonnest2 package in the R environment.

Table 5 shows results for the model comparisons considering Amplitude and Latency of N170. As for the previous analyses (see Tables 2, 3), four models were considered with respect to the four ROIs previously defined. Overall, the Vuong's test did not allow to reject the null hypothesis of indistinguishable between models with and without the Gender Variable. The model, in all ROI, showed very similar BICs. This strongly suggests that the evidence of the models is the same. Indeed, the 95% confidence intervals of ΔBIC overlapped the zero, implying that the models are enough close and M1s cannot be preferred over M0s. These results would suggest that including the gender variable in the models (M1s) did not improve their evidence with regards to the previous models (M0s). In this case, adding Gender Effect, don't significantly change the evidence of the model, when compared to the sample data. Therefore, using Occam's razor, we resorted to considering the simplest models in terms of parameters, according to the principle of simplifying the variables in an experiment (Srinagesh, 2006; Luck and Gaspelin, 2017).

**Table 5:** Comparison respect to gender effect.

**Gender effect**					
	**Model**	**BIC M0**	**BIC M1**	**Vuong's statistic**	**95% CIs** Δ**BIC**
N170 Amplitude	L-ANT	3432.45	3448.54	0.026 (*p* = 0.994)	[−35.38, 3.20]
	R-ANT	3160.68	3157.28	0.053 (*p* = 0.3)	[−24.47, 31.26]
	L-POST	2323.46	2345.85	0.026 (*p* = 0.998)	[−37.30, −7.47]
	R-POST	2460.68	2461.10	0.048 (*p* = 0.997)	[−21.13, 20.30]
N170 Latency	L-ANT	9825.47	9826.65	0.042 (*p* = 0.5)	[−26.18, 23.83]
	R-ANT	9703.89	9719.64	0.017 (*p* = 0.6)	[−31.77, 0.27]
	L-POST	7927.17	7929.67	0.05 (*p* = 0.5)	[−26.69, 21.71]
	R-POST	7696.23	7675.25	0.09 (*p* = 0.6)	[−11.63, 53.0]

*In the Table, M0 indicates the model without the gender variable whereas M1 the model where the gender variable was instead added. The Vuong's statistic considers the difference of two models in terms of log-likelihood. The statistic evaluates the hypothesis that the models M0 and M1 are indistinguishable with regards to the sample data. ΔBIC indicates the difference (M0-M1) in terms of their BIC values whereas 95% CIs are computed with the asymptotic formula*.

#### Decision-tree modeling: target and non-target stimuli

To validate that the emotional bias, generated by combined stimuli, is correlated with the class of participant (musicians/non-musicians), we processed the input data calculating, for each participant, the relative variation between the music conditions considering each EEG channel.

To do this, we used the following equation (Equation 1):

(1) $$\begin{array}{r} \Delta {\text{x}}_{ij}=\left|\frac{x_{k}-x_{a}}{x_{a}}\right| \end{array}$$

where *i* ∈ 1, …, 24 was the participant, *j* ∈ 1, …, 61 was the EEG channel, a = Albeniz; k = Chopin or Mozart.

The output data was further processed to evaluate which EEG channels showed the best discrimination capability for the classification between the two groups. A predictive model was implemented using a tree-building algorithm (Menolascina et al., 2007), by generating prediction rules from partially pruned decision trees that were built using C4.5 Quinlan's heuristics (Quinlan, 1993), whose main goal consists in the minimization of the tree levels and nodes number, thereby maximizing data generalization. This technique uses an information theoretical procedure to select, at each choice point in the tree, the attribute that would maximize the information gained from splitting the data.

#### Predictive model results

The predictive model was trained and tested 200 times considering different random combinations of training and test sets, obtained from the input dataset considering a splitting percentage of 81.82%. The results are expressed as mean values, considering 200 iterations, of Accuracy, Sensitivity, Specificity and Area Under the Curve (AUC) and are reported in Tables 6, 7.

**Table 6:** Mean performances of the predictive models – N170 amplitude.

**Face**	**Dataset**	**Accuracy %**	**Sensitivity**	**Specificity**	**AUC**
Fear	Albeniz vs. Mozart	32.75	0.28	0.38	0.31
	Albeniz vs. Chopin	**62.00**	0.52	0.73	0.72
Happy	Albeniz vs. Mozart	**66.88**	0.60	0.74	0.67
	Albeniz vs. Chopin	37.00	0.33	0.41	0.37
Neuter	Albeniz vs. Mozart	**63.50**	0.68	0.60	0.64
	Albeniz vs. Chopin	49.88	0.54	0.46	0.50

*These models highlight that in happy faces ERP amplitude is modulated as in neuter faces (in Albeniz vs. Mozart predictive model)*.

**Table 7:** Mean performances of the predictive models – N170 latency.

**Face**	**Dataset**	**Accuracy %**	**Sensitivity**	**Specificity**	**AUC**
Fear	Albeniz vs. Mozart	**64.50**	0.61	0.68	0.70
	Albeniz vs. Chopin	41.88	0.36	0.48	0.41
Happy	Albeniz vs. Mozart	**51.88**	0.47	0.57	0.53
	Albeniz vs. Chopin	**75.88**	0.70	0.81	0.76
Neuter	Albeniz vs. Mozart	42.88	0.41	0.45	0.41
	Albeniz vs. Chopin	**65.5**	0.46	0.85	0.66

These models highlight that in happy faces ERP latencies are modulated as in neuter faces (in Albeniz vs Chopin predictive model)

We tried to improve the performance of the previous predictive model by reducing the number of the considered EEG channels using a correlation-based filter that selects the most highly correlated features. A fast correlation-based filter (FCBF) algorithm (Yu and Liu, 2003) was adopted.

The same procedure discussed in the previous section was applied considering the obtained subset of EEG channels, and a new predictive model was implemented and evaluated.

The performance of the new predictive model is reported in Tables 8, 9.

**Table 8:** Mean performances of the FCBF-filtered predictive models – N170 amplitude.

**Face**	**Dataset**	**EEG channels**	**Accuracy %**	**Sensitivity**	**Specificity**	**AUC**
Fear	*Albeniz* vs. *Mozart*	–	–	–	–	–
	*Albeniz* vs. *Chopin*	P3–CPz	71.65	0.70	0.74	0.77
Happy	*Albeniz* vs. *Mozart*	T8–F4	69.27	0.64	0.74	0.72
	*Albeniz* vs. *Chopin*	–	–	–	–	–
Neuter	*Albeniz* vs. *Mozart*	CP1	79.13	0.69	0.8925	0.81
	*Albeniz* vs. *Chopin*	FCz–CPz	66.38	0.73	0.60	0.67

*In this amplitude dataset, we can see electrodes more sensible to predictive model. In Fear face, EEG channels are P3 and CPz, In Happy face T8 and F4 and in Neuter Face CP, FCz, and CPz*.

**Table 9:** Mean performances of the FCBF-filtered predictive models – N170 latency.

**Face**	**Dataset**	**EEG channels**	**Accuracy %**	**Sensitivity**	**Specificity**	**AUC**
Fear	*Albeniz* vs. *Mozart*	Fp1–AF7	79.75	0.84	0.76	0.80
	*Albeniz* vs. *Chopin*	F8	65.50	0.45	0.86	0.66
Happy	*Albeniz* vs. *Mozart*	CP5	61.13	0.94	0.45	0.69
	*Albeniz* vs. *Chopin*	CP1–C5–CPz–AF4	82.00	0.78	0.87	0.82
Neuter	*Albeniz* vs. *Mozart*	–	–	–	–	–
	*Albeniz* vs. *Chopin*	FC1–O2–F4–AF7–FT7–FT8–AF8	70.63	0.49	0.92	0.71

*In this latency dataset we can see electrodes more sensible to predictive model. In Fear face, EEG channels are Fp1, AF7 and F8; in Happy face CP5, CPP1, C5, CPz, and AF4 and in Neuter Face FC1, O2, F4, AF7, FT7, FT8, and AF8*.

## Discussion

Our aim was to investigate modulation of emotional face recognition by cross-modal perception, treated as a function of background music. Synesthesia and crossmodal perception can have a strong modulatory effect on cortical processing, conditioning or facilitating perception and interpretation of the administered stimulus. We analyzed how musicians' recognition of facial expressions was affected by music-induced emotions. These data allow us to suggest that the presence of emotional information from another sensory channel (i.e., auditory information from background music) activates cross-modal integration of information and that this process can be modulated by the perception of the musical stimulus. This salience, for emotional face, could be explicable in terms adaptive: identify more early stage emotions is a skill that, developmentally, can be crucial for the survival and, proximal and contingent, is an indispensable social competence (Niu et al., 2012). So, in a condition where the participants are more “emotionally involved,” the neuter face, that is ambiguous for a defined emotional recognition and that is more difficult to recognize, can be more affected by emotion music induced. This justifies the fact that the musicians evaluated music as more pleasant and emotional (happy and sad) than non-musicians, and this judgment on emotional engagement is in agreement with their musical appraisal and competence. This emotional involvement leads to a delay in reaction times. These results imply that the motor and perceptual systems can be modulated, in a top-down process, by music-induced emotions. The electrophysiological data revealed increased N170 amplitudes in musicians in all conditions. The background music had less impact in non-musicians, then can produce less bias in the task. Instead, an earlier onset of the global processing of the stimulus indicates that music interacts with the interpretation of salience, producing a behavioral delay and an increased cortical arousal in musicians. This result suggests that perception of facial expressions can vary according to perceptions of a concurrent auditory stimulus and an individual's musical background.

The decreased ERP amplitude, faster reaction times and lower VAS scores in the non-musicians group, suggests that non-musicians found the background music less engaging and emotionally arousing. Hence their top-down processes (less modulated by musical listening), doesn't bias the face perception. The relative changes in arousal, during the face recognition process, are driven by the subjective emotional reaction and top-down processing. The evidence of this concept was obtained from the comparison of responses to the neutral face (Target) whilst listening to music by Albeniz (pleasant), Mozart (happy) and Chopin (judged, at the same time, both sad and pleasant). We also assessed whether, within our model, there was a gender effect (Miles et al., 2016), but, in our study, gender analysis did not improve evidence with regards to the simpler model. In this case, adding gender effect, don't significantly change the evidence of the model, when compared to the sample data. We chose to keep the simpler model, even in accordance with the latest methodological ERP guidelines (Luck, 2005; Luck and Gaspelin, 2017). Probably in a future study, increasing the number of the sample, so that we can analyze the gender effect within the model, we could implement the complex model.

In view of these results, to investigate other possible bias variable-related, we sought to determine whether the bias effect could be present not only on neutral faces, as literature highlight. According to this hypothesis, we tested, using the predictive model, the N170 components for the other face emotional expressions showed during the task (happy and fear).

The predictive model allowed us to determine the most significant decision-tree features; in fact, the classification performances obtained using the trained predictive model were high, regardless of training and test sets. In this case, we find modulation of the response even in happy faces, but not in fear faces. This could also be explained by theories on emotions where the stimulus that produces fear is the least susceptible to alterations because it is the one most immediately and easily perceived (Vuilleumier et al., 2001; Phelps and LeDoux, 2005; Almeida et al., 2016).

Emotional salience allows the recognition and discrimination of neutral expressions. Our data indicate that the simultaneous presence of emotional information from multiple sensory channels activates a process of crossmodal integration that could be facilitated by music. Further research using different neuroscientific and behavioral techniques and paradigms is needed to improve our understanding of emotional crossmodal integration.

## Ethics statement

Lecce Ethical Comittee, ASL Hospital Vito Fazzi, approved the study with Verbal n. 2 all.21 approved the study in date October, 02, 2013.

## Author contributions

SI: Study design and coordination, whole data analysis design, manuscript preparation and editing and reviewing. AC: Statistical Data Analysis. AM: Subjects selection and data recording. GP: Subjects selections and data recording. RS: Subjects selection, Classical Music pieces choice. DT: Data recording. ID, AB, and VB: Machine Learning Data Analysis. MdT: Manuscript editing.

### Conflict of interest statement

The authors declare that the research was conducted in the absence of any commercial or financial relationships that could be construed as a potential conflict of interest.
